# Effects of trehalose and polyacrylate-based hydrogels on tomato growth under drought

**DOI:** 10.1093/aobpla/plac030

**Published:** 2022-07-02

**Authors:** Priera H Panescu, Marvin Browne, Kathleen K Chen, Lawren Sack, Heather D Maynard

**Affiliations:** Department of Chemistry and Biochemistry and California NanoSystems Institute, University of California, Los Angeles, 607 Charles E. Young Drive East, Los Angeles, CA 90095-1569, USA; Department of Ecology and Evolutionary Biology, University of California, Los Angeles, 621 Charles E. Young Drive South, Los Angeles, CA 90095-1569, USA; Department of Chemistry and Biochemistry and California NanoSystems Institute, University of California, Los Angeles, 607 Charles E. Young Drive East, Los Angeles, CA 90095-1569, USA; Department of Ecology and Evolutionary Biology, University of California, Los Angeles, 621 Charles E. Young Drive South, Los Angeles, CA 90095-1569, USA; Department of Chemistry and Biochemistry and California NanoSystems Institute, University of California, Los Angeles, 607 Charles E. Young Drive East, Los Angeles, CA 90095-1569, USA

**Keywords:** Drought, hydrogel, soil conditioner, tomato, trehalose

## Abstract

Hydrophilic amendments can enhance soil moisture content, which, in turn, can improve crop health under drought conditions. Understanding how different hydrogels interact with specific crops is necessary for optimal application. The soil conditioning abilities of a trehalose hydrogel and polyacrylate-based hydrogel were evaluated for tomatoes (*Solanum lycopersicum*) subjected to drought. Tomato plants were transplanted into individual pots with soil that contained trehalose hydrogel (0.4 wt%), polyacrylate-based hydrogel (0.4 wt%), or no hydrogel and subjected to a well-watered treatment or to pronounced soil drought, with or without rewatering. The health of tomato plants was monitored by measuring leaf total chlorophyll (a + b) concentration, leaf water potential (Ψ_leaf_), stomatal conductance (*g*_s_) and relative growth rate (RGR). The polyacrylate-based hydrogel, but not the trehalose hydrogel, improved tomato plant function under drought conditions, as indicated by improved *g*_s_ and RGR relative to the well-watered control. However, when subjected to a second drought, neither hydrogel was effective, and neither prolonged survival. The more hydrophilic polyacrylate-based hydrogel demonstrated promise in improving the growth of tomato plants under drought when included as a soil amendment at 0.4 wt%. This research is important for understanding the effects of these hydrogels as soil conditioners in drought prone systems.

## Teaser

Hydrophilic soil amendments are important to enhance soil moisture content during drought conditions. In this paper a trehalose hydrogel and a polyacrylate-based hydrogel were evaluated for tomato plants subjected to drought. It was shown that the polyacrylate-based hydrogel, but not the trehalose hydrogel, improved tomato plant function under drought conditions as indicated by improved stomatal conductance and relative growth rate compared to the well-watered control. This research is important to evaluate hydrogels that might prevent agricultural water wastage and improve the health of crops subjected to drought.

## Introduction

As drought frequency, severity and duration are exacerbated by climate change, improving the efficiency of water resources is crucial for a sustainable food supply (Food and Agriculture Organization of the United Nations (FAO) 2021). Drought negatively affects agriculture globally and, consequently, food security, water availability and rural livelihoods are negatively impacted as well. In the developing world alone, drought has caused $29 billion agricultural revenue loss between 2005 and 2015 (Conforti *et al.* 2018). Mitigation practices can negate the deleterious effects of drought-induced damage. During periods of drought, on-farm water and soil management have proven successful in bolstering crop productivity when faced with high temperatures and limited water (Earl and Davis 2003; Manickavelu *et al.* 2006; Farooq *et al.* 2009; Burke and Emerick 2016). Nonetheless, many water-intensive practices, such as flood irrigation, are still widely applied (MacDonald 2010). Technologies that prevent agricultural water wastage must be developed and implemented to improve the health of crops subjected to drought.

Hydrogels are hydrophilic polymeric materials capable of absorbing and releasing water many times their own mass (Buwalda *et al.* 2016). In soil, swollen hydrogels act as water reservoirs by slowly releasing captured water from the moist internal environment to the drier soil. Hydrogels have been mixed into soil to prevent water irrigation loss caused by drainage and evaporation (Akhter *et al.* 2004). They also offer a potential scaffold for controlled release of nutrients (Sannino *et al.* 2009; Teodorescu *et al.* 2009), and provide better oxygenation to plant roots by increasing soil porosity. By improving the water-holding capacity (WHC) of soil and water available to plant roots, hydrogels have demonstrated the ability to increase plant survival rate, water use efficiency and growth (Woodhouse and Johnson 1991; Abd El-Rehim *et al.* 2004; Akhter *et al.* 2004; Farrell *et al.* 2013; Orikiriza *et al.* 2013; Parvathy *et al.* 2014; Nassaj-Bokharaei *et al.* 2021). While superabsorbent polyacrylate gels have demonstrated success as soil conditioners, some have hypothesized that anionic moieties within hydrogels create electrostatic repulsions with negative charges on the surface of soil particles (Savi *et al.* 2014). The anion–anion repulsive forces can reduce adsorption of the hydrogel to soil and therefore allow the polymer to be leached by water over time. The development of alternative hydrophilic gels for soil conditioning could help overcome these issues and potentially demonstrate other advantages. The goal of this work is to investigate an alternative trehalose-based gel soil amendment for the maintenance of plant physiological function under drought.

The Maynard lab has developed a scalable, two-step synthesis of a trehalose-based hydrogel for the thermal stabilization of enzymes (Lee *et al.* 2015; Panescu *et al.* 2019). The synthesis yield was greatly improved from 17 to 88 %, scaled 100-fold while retaining a high yield at 76 % and optimized to eliminate the use of halogenated and toxic solvents. This multi-gram, green synthesis makes the gel more practical for agricultural applications where materials need to be cost-efficient and scalable (Kah *et al.* 2019). Moreover, trehalose can stabilize desiccant-intolerant soil bacteria necessary for plant growth (Gouffi *et al.* 1999; McIntyre *et al.* 2007). As such, trehalose hydrogels have potential for water management as well as stabilization and delivery of plant nutrients while being beneficial to soil. Here, we applied two hydrogels—the trehalose gel (Panescu *et al.* 2019) and a commercially available poly(acrylate-*co*-acrylamide)-based gel, Terra-sorb®—as soil amendments for tomato plants, *Solanum lycopersicum*, subjected to drought conditions. Performance of the gels was evaluated by monitoring tomato plant health and function through chlorophyll concentration, leaf water potential, stomatal conductance and relative growth rate (RGR) measurements.

## Materials and Methods

All growth experiments took place in a climate-controlled greenhouse in the Plant Growth Center at the University of California, Los Angeles (minimum, mean and maximum values for temperature, 20.2 °C, 22.7 °C, 27.1 °C; for relative humidity 38.8 %, 64.0 %, 79.3 %; and for irradiance 6.2, 177, 1326 μmol photons m^−2^ s^−1^; measured with a Hobo Weather Station, Onset, Bourne, MA, USA). Tomato seeds (*S. lycopersicum*; True Leaf Market, Salt Lake City, UT, USA) were sown in pots (7.95 cm width × 12.40 cm length × 5.87 cm deep) in soil (1:1:2:1:1 mixture of washed plaster sand, loam, peat moss, perlite, vermiculite). Note that the soil was not dried before adding hydrogel at 0.4 wt%. Terra-sorb® superabsorbent hydrogel was purchased from Gardener’s Supply Company (Burlington, VT, USA). Chlorophyll concentration was measured using Soil Plant Analysis Development (SPAD)-502 Chlorophyll meter (Spectrum Technologies, Aurora, IL, USA), which measures the difference in transmittance of red (650 nm) and infrared (940 nm) and gives readings that are correlated with total chlorophyll (a + b) concentration (Jiang *et al.* 2017). Leaf water potential (Ψ_leaf_) measurements were taken with a pressure chamber (0.001 MPa resolution, Plant Moisture Stress Model 1000; PMS Instruments Co.). Stomatal conductance (*g*_s_) was measured on the abaxial surface of given leaves using a porometer (AP-4, Delta-T Devices Ltd, Cambridge, UK).

### Trehalose hydrogel synthesis and characterization

#### Trehalose hydrogel synthesis.

 The hydrogel was synthesized at a multi-gram scale as described in a previous study (Panescu *et al.* 2019). Trehalose, a non-reducing disaccharide formed by α,α-1-1-linked glucose units, is first functionalized with vinyl groups and then cross-linked via redox-initiated polymerization with mono- and multifunctional trehalose acting as monomers and cross-linkers, respectively. Over 50 g of trehalose hydrogel was synthesized for these experiments.

#### Swelling ratio of trehalose hydrogel.

 Three individual trehalose gels were evaluated for their swelling ratio over 10 cycles of swelling and drying. For each cycle, hydrogels were dried for 24 h via lyophilization, then weighed (*M*_*D*_) before swelling them in Milli Q water over 72 h, then weighing the swollen gel (*M*_*S*_). The swelling ratios (*Q*_*M*_) were calculated as the mass ratio between the swollen gels and their initial dry mass.


$$\begin{array}{l} Q_{M}=\frac{M_{S}-M_{D}}{M_{D}} \end{array}$$

#### Evaluation of WHC of soil with different percentages of hydrogel.

 Terra-sorb® (0.4 wt%) or trehalose hydrogels (0.4 and 0.8 wt%) were mixed with dry soil (100 g). Controls without hydrogel were also prepared. All mixtures were weighed to obtain the soil dry mass (*M*_*D*_), then saturated with water for 24 h. The soils were then desaturated over 16 days and weighed every 2 days. All of the soils were rehydrated on Day 8. Experiments were conducted in triplicate. The WHC was calculated by using the soil mass at time point (*M*_*T*_) and the soil dry mass (*M*_*D*_):


$$\begin{array}{l} WHC=\frac{M_{T}-M_{D}}{M_{D}} \end{array}$$

### Terra-sorb® hydrogel: *S. lycopersicum* planting, soil amendment and water treatments

#### Experimental Set-up.

Seeds of *Solanum* were sown in pots (7.95 cm width × 12.40 cm length × 5.87 cm deep) in soil (1:1:2:1:1 mixture of washed plaster sand, loam, peat moss, perlite, vermiculite) and watered every other day. After ~2 weeks, at which time two sets of leaves had emerged and fully developed, the plants were thinned to one individual per pot with 1 kg of soil containing 0.4 wt% or 0.0 wt% (untreated control) hydrogel and watered regularly to keep soil moist for 2 weeks. At that time, five control plants were harvested and dried in a drying oven for at least 48 h at 75 °C until mass stabilized for RGR determination. The remaining pots were watered to full saturation and plants were measured with a chlorophyll meter. Five plants were then subjected to each of the following treatments over the course of 25 days: non-drought, i.e. watering to saturation each second day (ND); one drought, i.e. 9 days of suspended watering, followed by rewatering every second day for recovery for 7 days; or two droughts, i.e. 9 days of suspended watering followed by rewatering every second day for recovery for 7 days followed by another 9 days of suspended watering. We noted any major leaf wilting and leaf colour change. At the end of the experiment, the oven-dried mass of the dried leaves, stems and roots was measured.

#### Determination of chlorophyll concentration.

 Chlorophyll concentrations (SPAD) of the tomato plant leaves were determined for one leaf per plant (*n* = 5 plants per treatment).

#### Measurement of stomatal conductance and leaf water potential.

To determine the influence of the treatments on leaf-level gas exchange and plant water status, leaf water potential (Ψ_leaf_) measurements were paired with measurements of stomatal conductance (*g*_s_) for one leaf per individual plant (*n* = 5 plants per treatment). Leaf water potential represents the driving force for water within the tissue relative to pure water. As a plant dehydrates, leaf water potential becomes more negative as the volume of cell sap decreases, turgor pressure declines and solutes become more concentrated (Taiz and Zeiger 2014). A porometer (AP-4, Delta-T Devices Ltd, Cambridge, UK) measurement of *g*_s_ was taken once stable repeated values were achieved for each leaf before harvesting that leaf for water potential measurement; these measurements were conducted before the first drought was imposed, after the drought, after recovery and after the second drought period. Leaves were excised at the base of the petiole and sealed in sealable bags (Whirl-Pak, Madison, WI, USA), which had been exhaled into, to generate a high CO_2_, moist environment that minimized transpiration, then placed into a dark plastic bag with wet paper towels. Leaves were allowed to equilibrate within the bags for 30 min. Ψ_leaf_ was then determined for each leaf using a pressure chamber (0.001 MPa resolution, Plant Moisture Stress Model 1000; PMS Instruments Co.).

#### RGR measurements.

 After the tomato plants were thinned to one per pot and watered regularly for 2 weeks, and before the plants were subjected to separate conditions, five control plants were harvested (*t*_1_). The entire individual was gently disinterred, and the soil was removed carefully to preserve as many of the roots as possible. The plant roots, stems and leaves (no fruits and flowers were observed at this time) were separated and dried for at least 48 h at 70 °C before weighing (*M*_1_). After the plants were subjected to the different treatments for 25 days (*t*_2_), these plants were harvested in the same manner, and the roots, stems, leaves, flowers and fruit were separated and dried for at least 48 h at 70 °C before weighing (*M*_2_). Relative growth rate was calculated as:


$$\begin{array}{l} RGR=\frac{ln\;M_{2}\;\;\;-\;\;\;ln\;\;\;M_{1}\;\;\;}{t_{2}-t_{1}} \end{array}$$

### Trehalose hydrogel: *S. lycopersicum* planting, soil amendment and water treatments

The experimental set-up for this experiment was similar to that described for the Terra-sorb® hydrogels above. The only differences were: (i) the droughts lasted 11 days and (ii) SPAD, Ψ_leaf_ and *g*_s_ measurements were each taken a total of four times: at initial harvest, after the first drought phase, after the plants were allowed to recover and after a secondary drought phase.

### Statistical analysis

To test for an association between growth, drought treatments and the addition of gels, we performed ANOVAs for commercial and trehalose gels. For the commercial treatment, stomatal conductance was measured only once, on Day 25, so there is only the test of association for this stromal conductance data point between drought treatment and the presence of the gel. ANOVAs were performed using the *aov* function for the R statistical program (version 4.1.2). We then performed *post hoc* Tukey tests on significant models using the *TukeyHSD* function for R (version 4.1.2) **[see****Supporting Information—Table S1****]**. For repeated measurements of stomatal conductance, leaf water potential and SPAD, we performed a linear mixed effects model using *lmer* function of lme4 package for R (version 1.1-28). Models tested for the effect of treatment, presence of gels and their interactions with time **[see****Supporting Information—Table S2****]**.

## Results

### Evaluation of trehalose hydrogel properties

**Figure 1.**
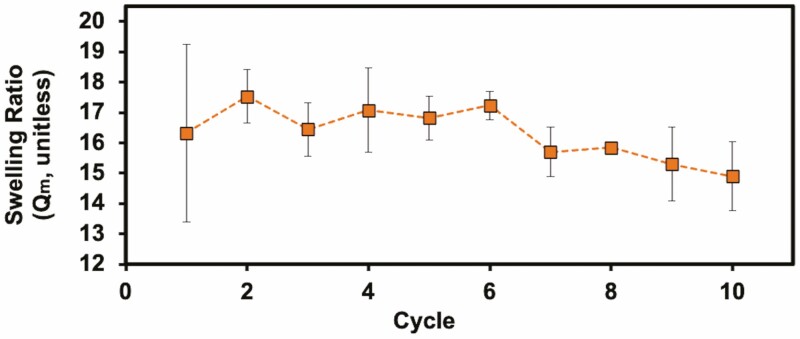
The swelling ratio of trehalose hydrogel over 10 cycles (40 days total) of dehydration and hydration shows less than a 9 % loss in mass (*n* = 3).

After purification and lyophilization, the trehalose hydrogels were swollen to their maximum capacity in 72 h in deionized water. This drying–swelling cycle was repeated where the dry mass was taken after lyophilization, and swollen mass was taken after swelling the gel in deionized water. The swelling ratio was calculated for each cycle by dividing the difference between the gels’ swollen mass and dry mass by the dry mass (Fig. 1). Over the course of 10 drying–swelling cycles (40 days total), the hydrogel swelling ratio decreased from 16.3 ± 2.9 to 14.9 ± 1.1 (8.8 % loss).

**Figure 2.**
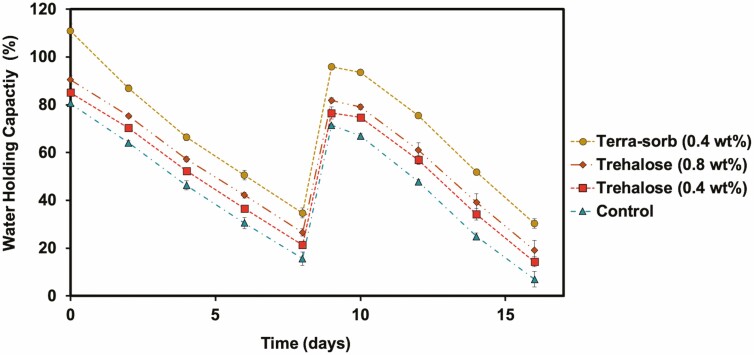
The variation in WHC of soil with different percentages of hydrogel over 16 days. Soil was saturated with water on Day 0 and Day 8 (*n* = 3).

To evaluate the WHC of sandy loam soil with hydrogel amendments, we applied Terra-sorb® at the manufacture’s recommended concentration, 0.4 wt%, and trehalose hydrogel at 0.4 wt% and 0.8 wt% in sandy loam soil. We saturated the soil, then allowed it to dehydrate over 8 days while monitoring water loss by mass (Fig. 2). All of the amendments demonstrated a statistically significant improvement in the WHC of the soil over the entirety of the experiment (e.g. hydrogel- amended soil versus control WHC demonstrated *P* < 0.05; Student’s *t*-test). Consistently, soil amended with Terra-sorb® gels (0.4 wt%) had the highest WHC, followed by soil with trehalose hydrogel at 0.8 wt% and then 0.4 wt%. We then rehydrated the soils to evaluate the capacities of the gels to retain their WHC through multiple drying cycles. The trehalose hydrogel applied at 0.8 wt% showed greater improvement to soil WHC relative to 0.4 wt% before the soils were rehydrated on Day 8. However, after rehydration, the soil WHC of the two concentrations did not statistically differ (e.g. soil amended with trehalose hydrogel at 0.4 wt% versus 0.8 wt% demonstrated *P* > 0.05; Student’s *t*-test). Otherwise, the conditioners maintained their previous trends and most of their WHC percentages.

### Effects of Terra-sorb® hydrogel on water-stressed tomato plants

In controlled greenhouse conditions, we grew tomato plants (*S. lycopersicum*) from seeds for 2 weeks and then thinned the plants to one individual per pot where pots either contained soil treated with 0.4 wt% hydrogel mixed in the top half of the soil, near the plant root zone, or were untreated controls. We continued watering the plants regularly for 2 weeks to ensure plant growth before beginning differential watering treatments. Individual pots were subjected to one of three conditions: watering to saturation each second day (ND and ND-G); one drought, i.e. 9 days of suspended watering, followed by rewatering every second day for recovery for 7 days (OD and OD-G); or two droughts, i.e. 9 days of suspended watering followed by rewatering every second day for recovery for 7 days followed by another 9 days of suspended watering (TD and TD-G) where ‘-G’ denotes a treatment with gel amendment. Here, ‘drought conditions’ were simulated by suspending watering while ‘normal watering conditions’ and ‘recovery’ were applied by watering every other day.

**Figure 3.**
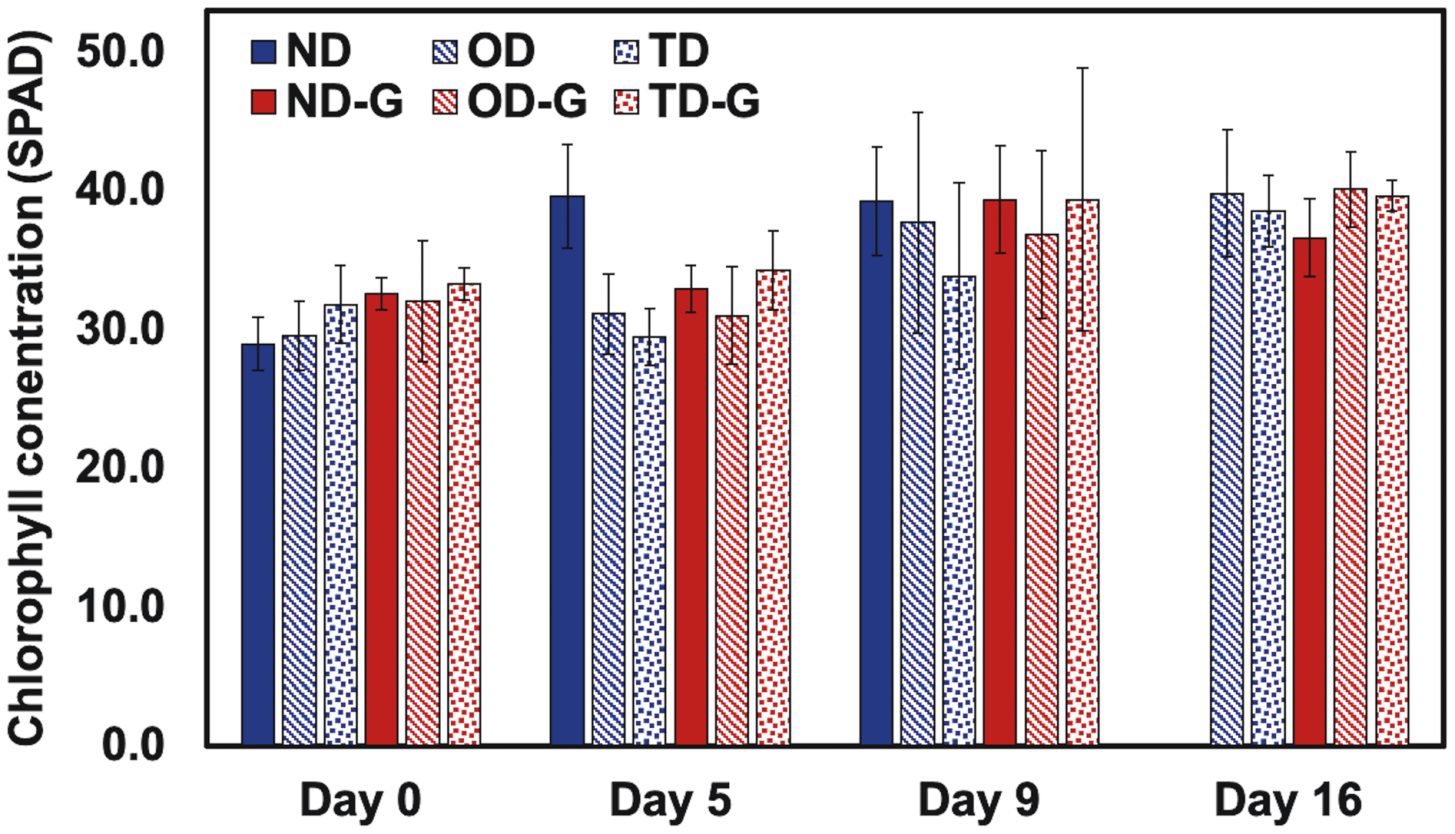
The chlorophyll concentration of *S. lycopersicum* leaves at various water treatments without (0.0 wt%) or with (0.4 wt%) Terra-sorb® superabsorbent hydrogel over 16 days. Normal watering conditions (ND and ND-G), conditions subjected to drought for 9 days followed by a recovery for 7 days (OD and OD-G) and drought for 9 days followed by a recovery for 7 days, followed by another drought for 9 days (TD and TD-G) are represented here (*n* = 5).

**Figure 4.**
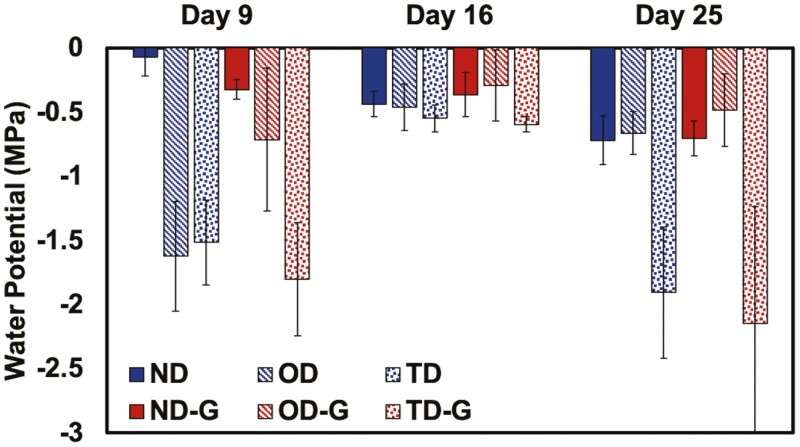
Leaf water potential (Ψ_leaf_) of *S. lycopersicum* in different water treatments without (0.0 wt%) or with (0.4 wt%) Terra-sorb® superabsorbent hydrogel over 25 days. Note the first drought ended on Day 9 and was followed by a recovery until Day 16 when TD and TD-G were subjected to a second drought. Normal watering conditions (ND and ND-G), conditions subjected to drought for 9 days followed by a recovery for 7 days (OD and OD-G) and drought for 9 days followed by a recovery for 7 days, followed by another drought for 9 days (TD and TD-G) are represented here (*n* = 5).

**Figure 5.**
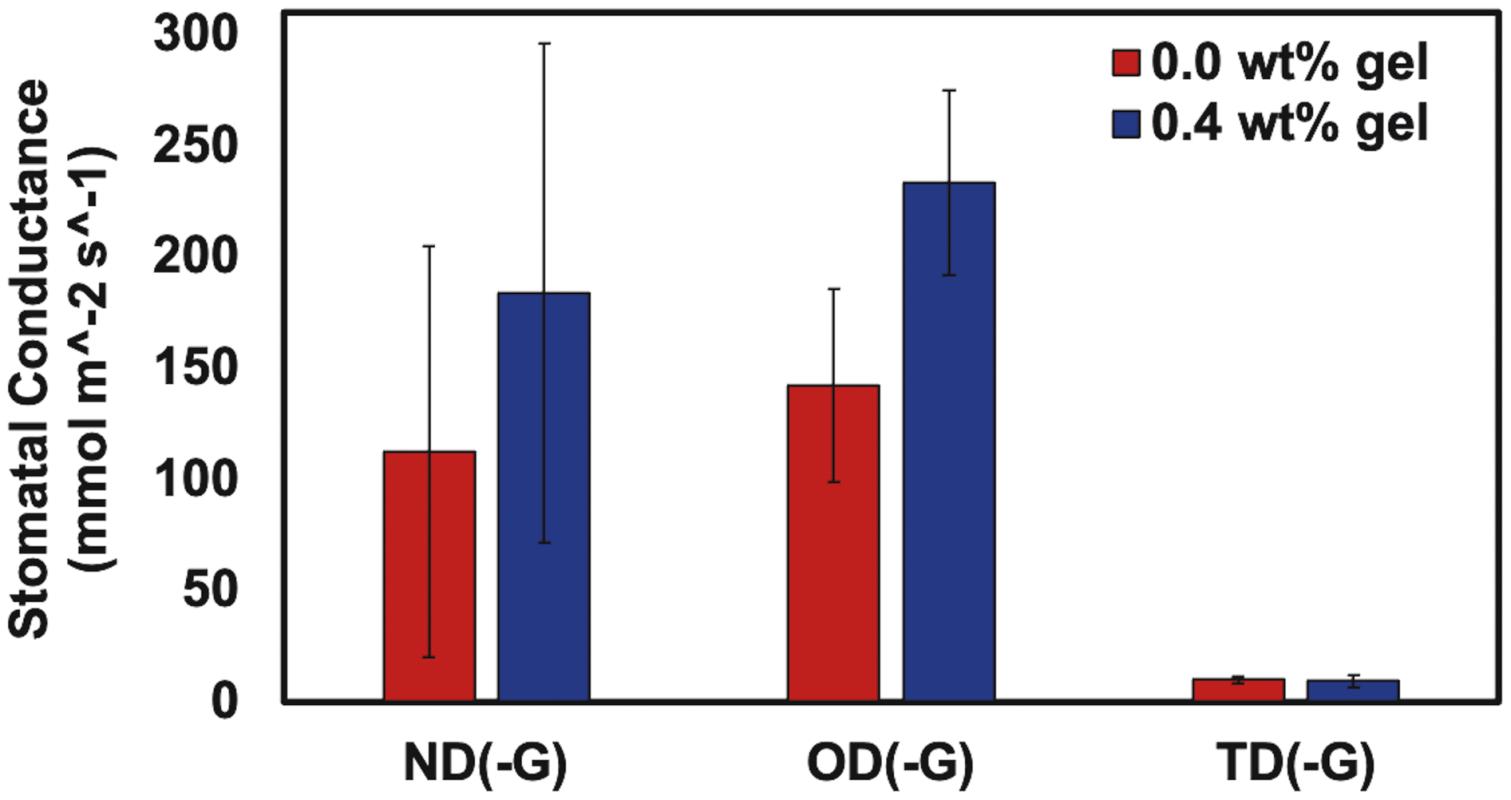
The stomatal conductance of *S. lycopersicum* after the various water treatments without (0.0 wt%) or with (0.4 wt%) Terra-sorb® superabsorbent hydrogel. Measurements were taken on Day 25 after both droughts were completed. Normal watering conditions (ND(-G)), conditions subjected to drought for 9 days followed by a recovery for 7 days (OD(-G)) and drought for 9 days followed by a recovery for 7 days, followed by another drought for 9 days (TD(-G)) are represented here (*n* = 5).

Throughout the experiment, measurements were taken to monitor plant function, that is, chlorophyll concentration (SPAD), leaf water potential (Ψ_leaf_) and stomatal conductance (*g*_s_). We evaluated the change in SPAD for the tomato plants on Day 0, 5, 9 and 16 of the experiment (Fig. 3). We generally observed no significant differences in chlorophyll concentration between the treatments whether the plants were watered or not, or whether in soil with or without hydrogel. The Ψ_leaf_ was measured on Day 9, 16 and 25 and declined during droughts and increased after recovery, but we observed no differences in Ψ_leaf_ between the treatments with or without hydrogel (Fig. 4), demonstrating that Terra-sorb® hydrogel has no effect on Ψ_leaf_. Stomatal conductance was measured on Day 25 and significantly declined for plants that underwent drought and recovered after watering. Additionally, for plants that underwent one drought cycle (OD), we observed a lower stomatal conductance in plants that were not treated compared to those treated with 0.4 wt% Terra-sorb® hydrogel (Fig. 5). The stomatal conductance of the plants that underwent two droughts was low and indistinguishable whether gel was present or not.

**Figure 6.**
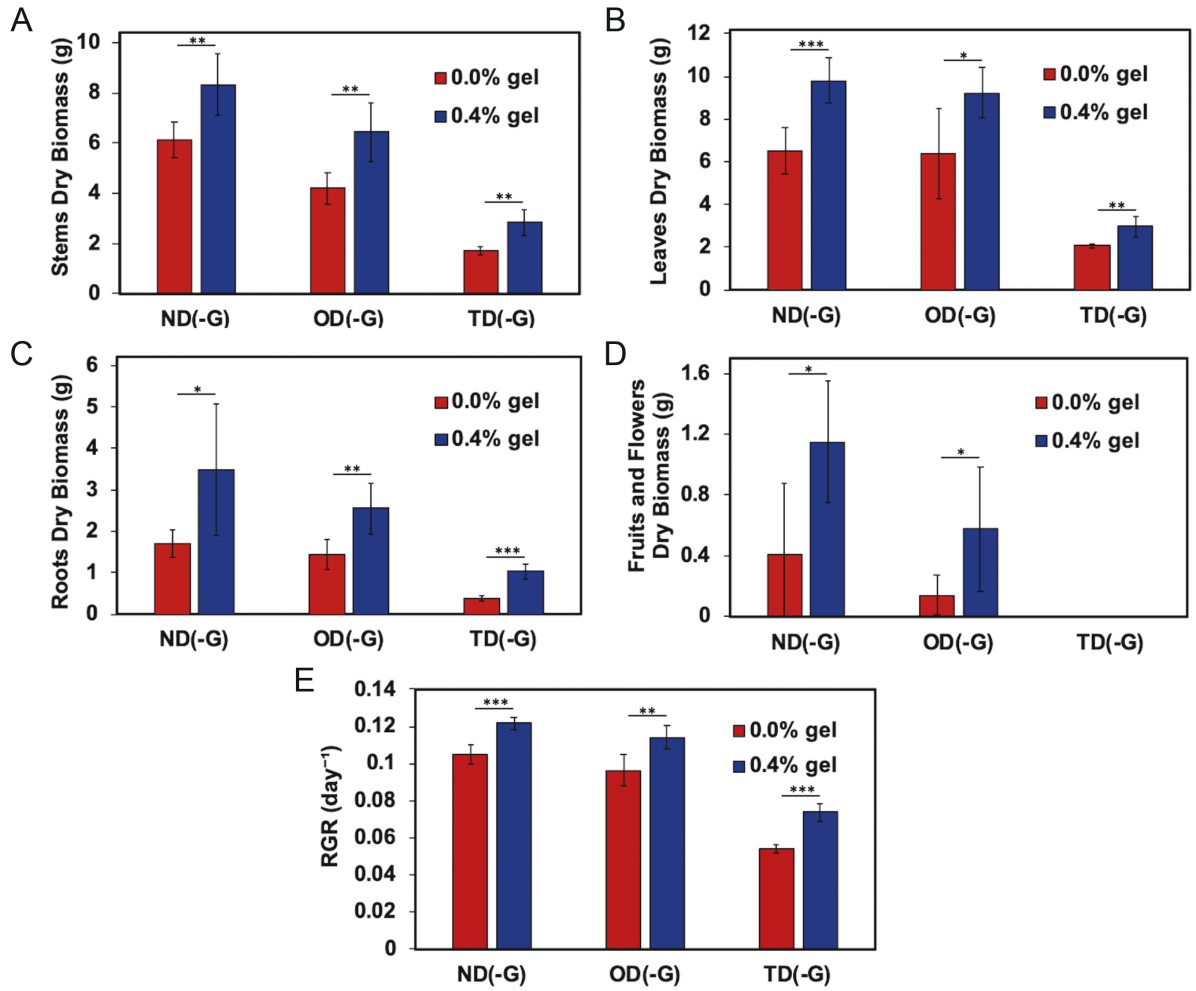
Dry biomass of *S. lycopersicum* (A) stems, (B) leaves, (C) roots and (D) fruits and flowers as well as the (E) RGR at various water treatments without (0.0 wt%) or with (0.4 wt%) Terra-sorb® superabsorbent hydrogel over 25 days. Normal watering conditions (ND(-G)), conditions subjected to drought for 9 days followed by a recovery for 7 days (OD(-G)) and drought for 9 days followed by a recovery for 7 days, followed by another drought for 9 days (TD(-G)) are represented here (*n* = 5). *P < 0.05; **P < 0.001.

**Figure 7.**
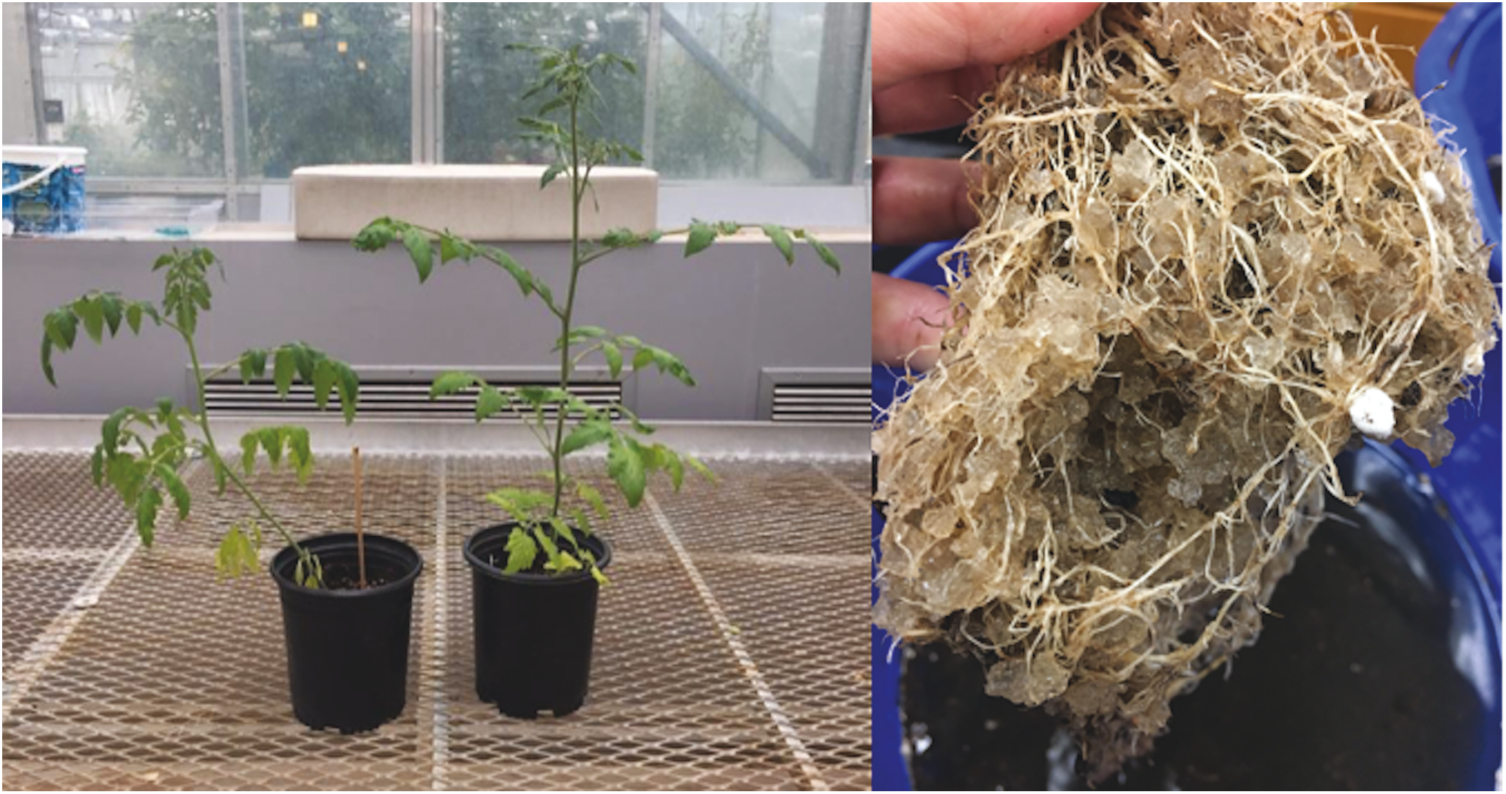
Visual representation of *S. lycopersicum* (left image) without (left) or with (right) Terra-sorb® superabsorbent hydrogel after one drought and root penetration into Terra-sorb® superabsorbent hydrogel (right image).

At the end of the experiment, plants were analysed for dry mass and RGR. For all watering conditions, we observed significant increases in the dry mass of tomato stems (Fig. 6A), leaves (Fig. 6B) and roots (Fig. 6C) when the hydrogel was present. Additionally, in regular watering conditions ND(-G), tomato plants grown in soil treated with hydrogel demonstrated a higher RGR of fruits and flowers compared to the control (Fig. 6D). However, for conditions OD(-G) and TD(-G), where drought(s) were applied, fruits and flowers were not observed on any plants that were subjected to two droughts. Relative growth rate was also improved by the addition of Terra-sorb® hydrogel for all treatments (Fig. 6E). A visual representative of OD and OD-G before harvesting can be seen in Fig. 7.

### Effects of trehalose hydrogel on water-stressed tomato plants

**Figure 8.**
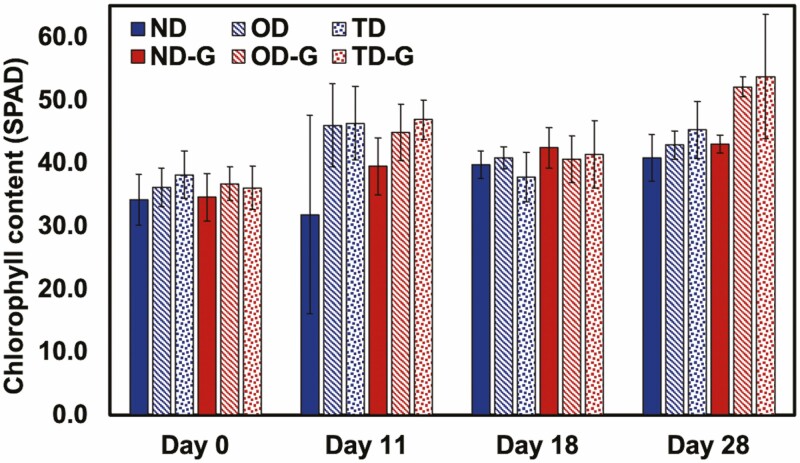
The chlorophyll concentration of *S. lycopersicum* leaves at various water treatments without (0.0 wt%) or with (0.4 wt%) trehalose hydrogel over 28 days. Normal watering conditions (ND and ND-G), conditions subjected to drought for 11 days followed by a recovery for 7 days (OD and OD-G) and drought for 11 days followed by a recovery for 7 days, followed by another drought for 10 days (TD and TD-G), where ‘-G’ denotes a treatment with gel amendment, are represented here (*n* = 5).

**Figure 9.**
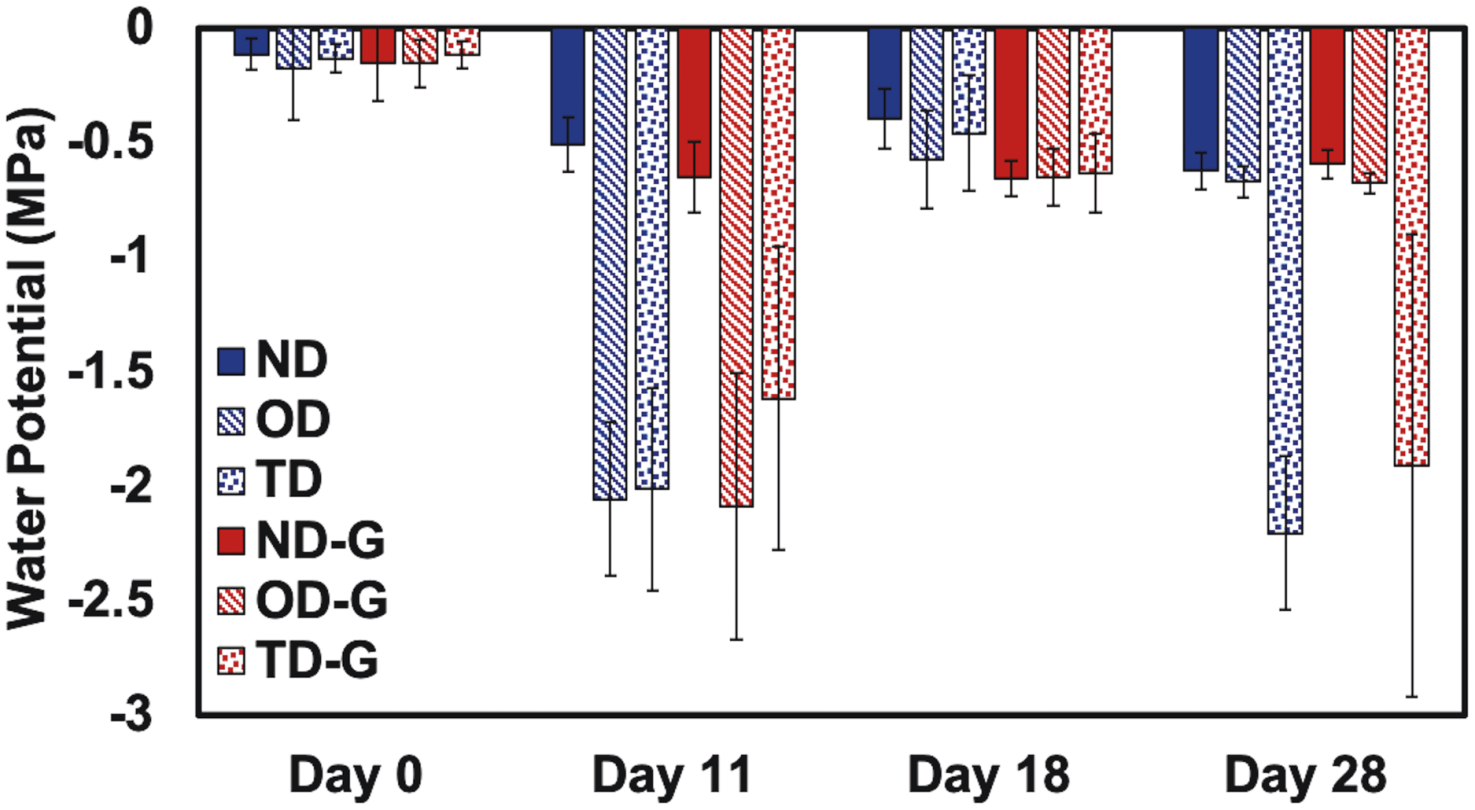
Leaf water potential (Ψ_leaf_) of *S. lycopersicum* at various water treatments without (0.0 wt%) or with (0.4 wt%) trehalose hydrogel over 28 days. Normal watering conditions (ND and ND-G), conditions subjected to drought for 11 days followed by a recovery for 7 days (OD and OD-G) and drought for 11 days followed by a recovery for 7 days, followed by another drought for 10 days (TD and TD-G), where ‘-G’ denotes a treatment with gel amendment, are represented here (*n* = 5).

**Figure 10.**
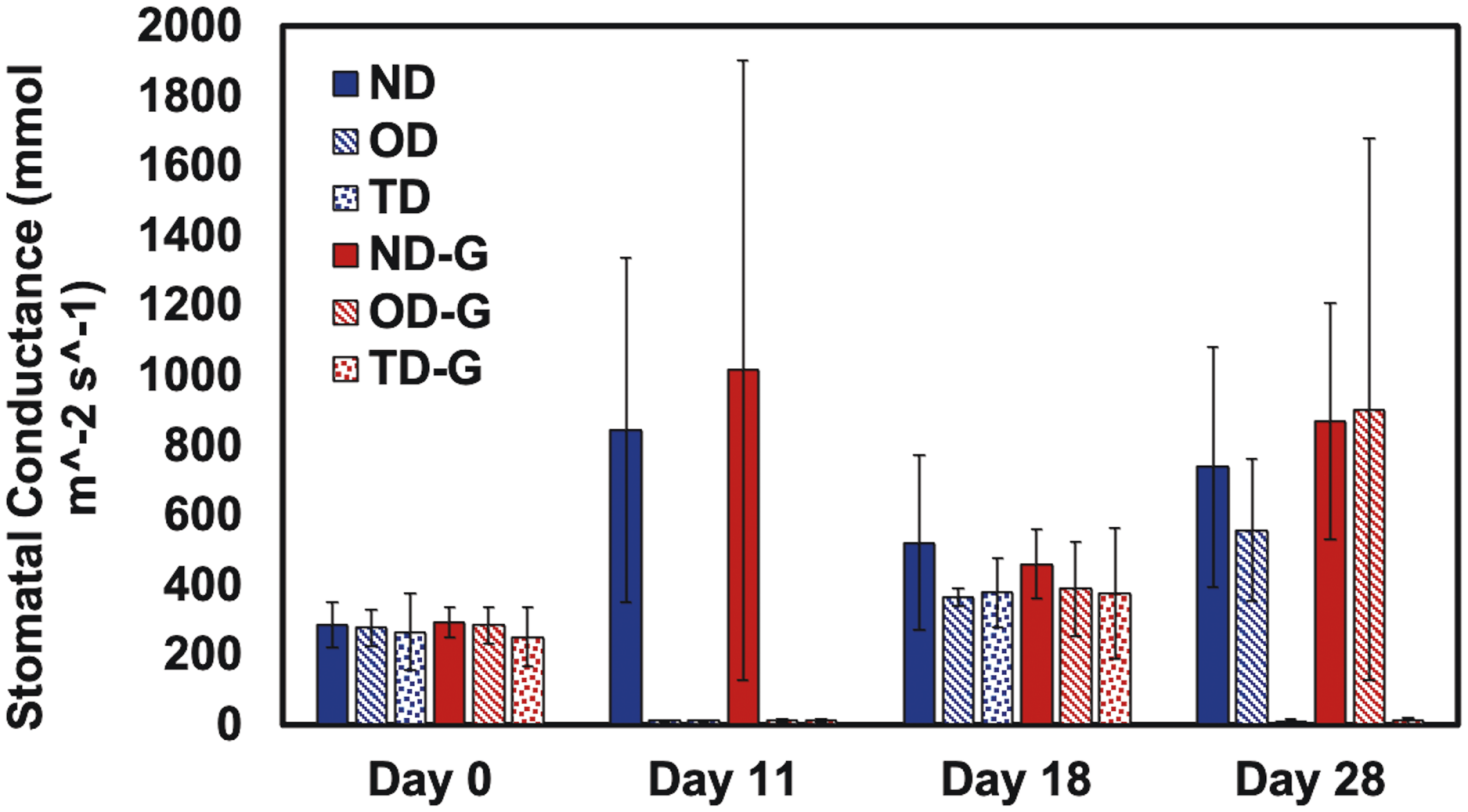
The stomatal conductance of *S. lycopersicum* after the various water treatments without (0.0 wt%) or with (0.4 wt%) trehalose hydrogel. Normal watering conditions (ND and ND-G), conditions subjected to drought for 11 days followed by a recovery for 7 days (OD and OD-G) and drought for 11 days followed by a recovery for 7 days, followed by another drought for 10 days (TD and TD-G), where ‘-G’ denotes a treatment with gel amendment, are represented here (*n* = 5).

**Figure 11.**
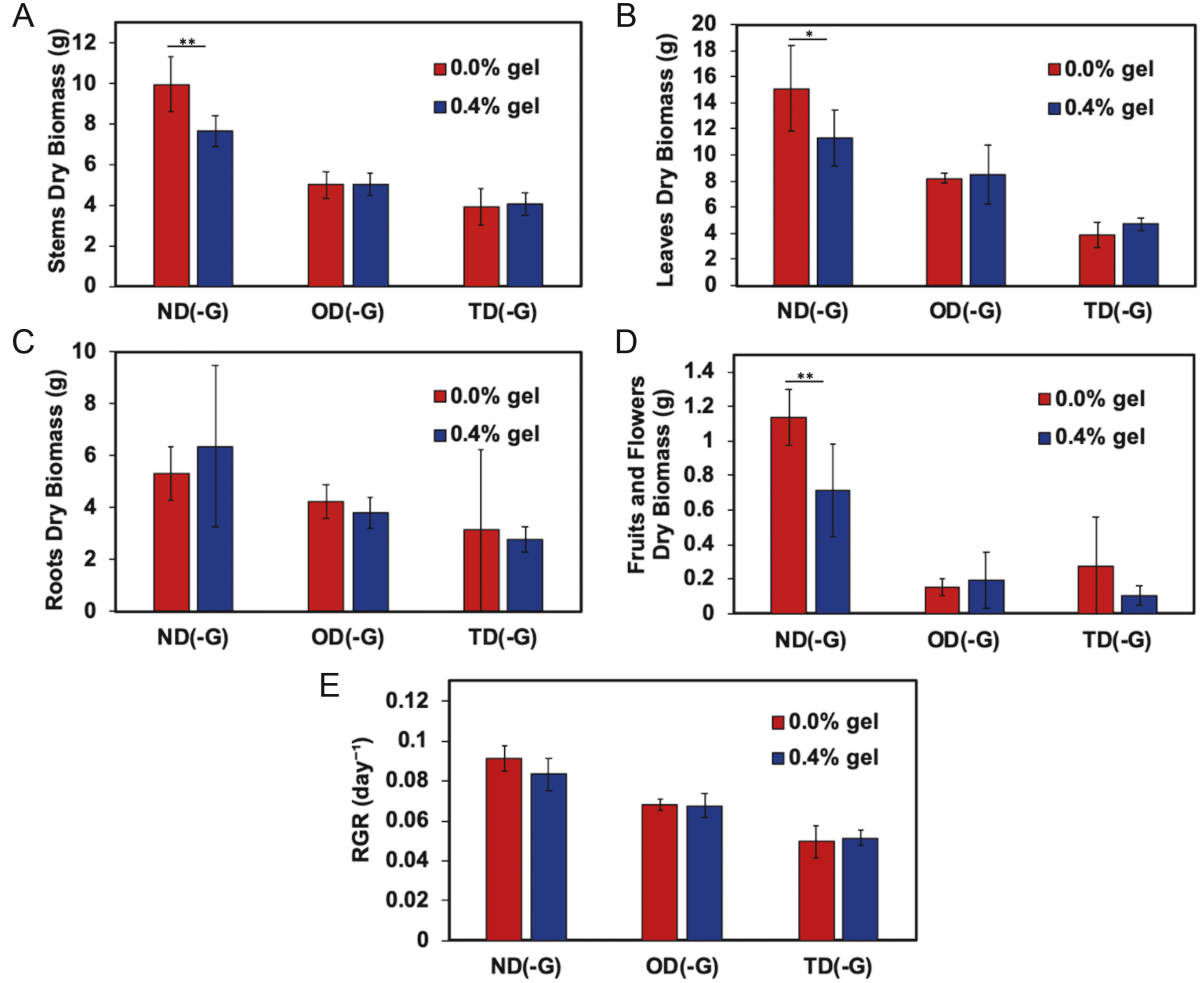
Dry biomass of *S. lycopersicum* (A) stems, (B) leaves, (C) roots and (D) fruits and flowers as well as (E) RGR at various water treatments without (0.0 wt%) or with (0.4 wt%) trehalose hydrogel over 28 days. Normal watering conditions (ND(-G)), conditions subjected to drought for 11 days followed by a recovery for 7 days (OD(-G)) and drought for 11 days followed by a recovery for 7 days, followed by another drought for 10 days (TD(-G)) are represented here (*n* = 5). *P < 0.05; **P < 0.001; ***P < 0.005.

**Figure 12.**
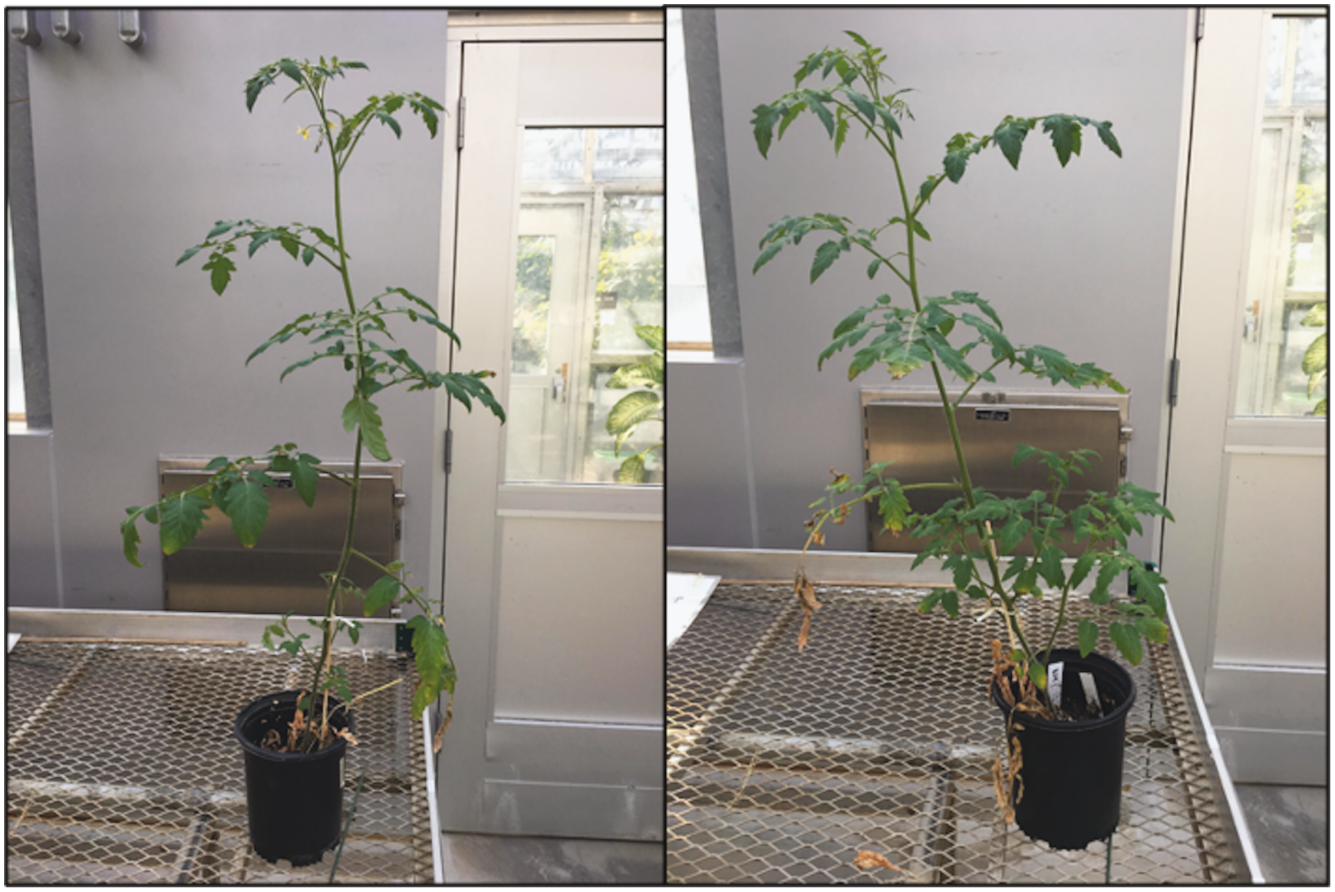
Visual representation of *S. lycopersicum* without (left) or with (right) trehalose hydrogel after two droughts.

Using similar experimental conditions as described above, we applied trehalose hydrogel also at a 0.4 wt% concentration (with respect to the soil mass, mixed into the top half of the soil near the root zone). The following changes were made from the previous experiment: (i) the droughts both lasted for 11 days and (ii) SPAD, Ψ_leaf_ and *g*_s_ measurements were collected at slightly different time points; all were collected on Days 0, 11, 18 and 28. Overall, there were no statistical differences in the SPAD (Fig. 8), Ψ_leaf_ (Fig. 9), *g*_s_ (Fig. 10) or growth measurements (Fig. 11), between the plants with or without gel under the same treatment. Plants that underwent two droughts TD(-G) are shown in Fig. 12. The only statistical differences were between treatments that were watered and those subjected to drought.

## Discussion

### Evaluation of trehalose hydrogel properties

Due to climate change, water availability has become more sporadic, causing plants to experience increasing frequencies of drought and rewatering cycles (Xu *et al.* 2010). As hydrogels may be used to ameliorate plant water status during such cycles, we tested the ability of the trehalose hydrogels to retain their swelling ratio through repeated drying and wetting cycles. The minimal loss (<9 wt%) in swelling ratio during these cycles is an indicator that the gel can be subjected to multiple drought cycles while largely retaining its swelling abilities.

Next, we evaluated how the WHC of a sandy loam soil was affected by Terra-sorb® and the trehalose hydrogel. Although all hydrogels improved the WHC of the soil, Terra-sorb® (0.4 wt%) demonstrated the most improvement to the WHC, likely due to its more hydrophilic structure. While WHC is an important factor for soil quality, the water held by hydrogels is not necessarily available to crops (Farrell *et al.* 2013). As such, it is vital to also monitor plant growth in soil with the hydrogel amendments.

### Effects of Terra-sorb® hydrogel on water-stressed tomato plants

Previous studies have demonstrated that hydrogel soil conditioners are not always effective in sustaining plant health and growth, and, in fact, are sometimes detrimental, depending on the soil type, plant species and experimental conditions (McGuire *et al.* 1978; Wang and Boogher 1987; Akhter *et al.* 2004). Thus, before testing trehalose hydrogels directly, we ensured that tomato plants and our simulated drought conditions could benefit from soil conditioners by using commercially available hydrogel, Terra-sorb® at 0.4 wt%, that has previously demonstrated delayed moisture loss for *Quercus ruba* seedlings subjected to short-term dehydration stress (Apostol *et al.* 2009). We included treatments with drought recovery and a second drought to evaluate the effect of the hydrogels on the tomato plants’ resilience to drought-recovery cycles, as typically plants rewatered after drought can show stomatal re-opening, photosynthetic recovery and renewed growth (Xu *et al.* 2010). The recovery conditions of crops after drought heavily determine the long-term impact on plant health where wetter conditions typically expedite recovery (Schwalm *et al.* 2017). Understanding how hydrogels affect recovery is therefore vital to assessing their potential as conditioners.

Chlorophyll concentration can be used as an indicator of allocation to light harvesting (Netto *et al.* 2005; León *et al.* 2007; Fu *et al.* 2013). In drought conditions, plants often experience reductions in chlorophyll concentration (Anjum *et al.* 2003). Here, drought and Terra-sorb® hydrogel had no significant influence on leaf chlorophyll concentration.

Next, to determine the extent of the drought on the plant water status after the first drought, we evaluated the leaf water potentials (Ψ_leaf_), whereby a lower value indicates greater leaf dehydration (Siddique *et al.* 2000; Atteya *et al.* 2003). While water potential decreased in response to drought, there were no notable effects from the hydrogel.

The stomatal conductance (*g*_s_) of the tomato plant leaves was paired with Ψ_leaf_ measurements. The stomata are microvalves on leaf surfaces that facilitate the gas exchange of carbon dioxide and water between plants and the atmosphere (Sack *et al.* 2006; Henry *et al.* 2019). Stomatal opening enables leaf photosynthesis, plant growth and water use, whereas stomatal closure is necessary for plant survival during drought. This regulation is crucial for plants as a majority of loss from transpiration is through stomatal opening (Wan *et al.* 2009). As most leaf water exchange occurs through stomatal pores, drought stress causes leaf stomatal closure and therefore reduced stomatal conductance (Brodribb and Holbrook 2003; Egilla *et al.* 2005; Kawakami *et al.* 2006; Henry *et al.* 2019). As indicated by the enhanced stomatal conductance of plants in soil with hydrogel, Terra-sorb® did help increase drought tolerance in the tomato plants subjected to one drought. The extremely low stomatal conductance of all plants subjected to two droughts may be attributed to irreversible tight closure of the stomatal pores; stomatal pores would reopen once leaves are rehydrated only if there is not irreversible cellular damage (Trueba *et al.* 2019).

Lastly, we compared the final dry biomass of all conditions as water stress can impose negative effects on crop yield (Nam *et al.* 2001; Samarah 2005). Specifically, RGR represents the exponential mass increase relative to the length of time between measurements (Wright *et al.* 2004). Terra-sorb® amendments enhanced tomato health as indicated by the improved RGR of tomato stems, leaves, roots, fruits and flowers. We also observed root penetration of hydrogels (Fig. 7) which could be due to drought stress but has also been previously suggested to increase plant-available water in studies that applied hydrogels to lettuce and barley seedlings (Woodhouse and Johnson 1991) and tree cuttings (Shi *et al.* 2010). Overall, Terra-sorb® at 0.4 wt% in sandy loam soil demonstrates promise as an agricultural amendment that can improve tomato plant health significantly.

### Effects of trehalose hydrogel on water-stressed tomato plants

While the trehalose hydrogel did not have any significant effects on tomato plants at the applied concentration, the hydrogels may be effective at higher concentrations or in different environmental conditions, such as a soil with higher sand composition. We hypothesize that the trehalose hydrogel was not as effective as Terra-sorb® because it does not absorb water as well. The hydrophobic styrenyl backbone of the hydrogel prevents high swelling ratios, so it was not effective as a soil conditioner in the treatments described in this report. It should be noted that, to our knowledge, the gels did not negatively affect plant growth, as demonstrated in previous studies with other gels (Agaba *et al.* 2010). As such, trehalose hydrogels could be applied for another agricultural application such as bacteria stabilization since trehalose is known to stabilize desiccant-intolerant soil bacteria necessary for plant growth (Gouffi *et al.* 1999; McIntyre *et al.* 2007). Additionally, the trehlose hydrogels were shown to improve the thermostability of various enzymes (Panescu *et al.* 2019) and could be tested for its ability to stabilize exogenous enzyme supplements to soil (Shackle *et al.* 2006).

## Conclusion

Trehalose and Terra-sorb® hydrogels were tested for their ability to improve physiological properties of tomato plants subjected to drought(s). While the Terra-sorb® hydrogel did improve the dried biomass yields and stomatal conductance of tomato plants subjected to drought, the trehalose hydrogels had little to no effect on plant growth under any water conditions. At the tested concentration, the trehalose hydrogels were not effective soil conditioners, but could potentially be used for other agricultural applications as they had no detectable detrimental effects on the health of the tomato plants.

## Supporting Information

The following additional information is available in the online version of this article—

**Table S1**. Results of two-way ANOVAs estimating the effect of drought treatments and presence of either commercial or trehalose gels in the soil on growth and organ allocation.

**Table S2.** Results of linear mixed models estimating the effect of drought treatments and presence of either commercial or trehalose gels in the soil on leaf water potential, SPAD and stomatal conductance with time.

plac030_suppl_Supplementary_Material

## Data Availability

Primary data for this work can be found at this site: https://github.com/brownegm/Panescu_etal2022.

## Notes

**Plants, Ecosystems & Climate. Chief Editor**: Mary Heskel
