# Supplementary material for: Effects of trehalose and polyacrylate-based hydrogels on tomato growth under drought
Source: AoB Plants. 2022 Jul 2;14(4):plac030. doi: 10.1093/aobpla/plac030 (PMC9330256; doi:10.1093/aobpla/plac030)
Supplement: plac030_suppl_Supplementary_Material [file plac030_suppl_supplementary_material.pdf]

Supporting Information

Effects of Trehalose and Polyacrylate-Based Hydrogels on Tomato Growth Under Drought

Priera H. Panescu [1†], Marvin Browne [2†], Kathleen K. Chen[1], Lawren Sack[2*], and Heather D. Maynard[1*]

[1]Department of Chemistry and Biochemistry and California NanoSystems Institute, University of California, Los Angeles, 607 Charles E. Young Drive East; [2]Department of Ecology and Evolutionary Biology, University of California, Los Angeles, 621 Charles E. Young Drive South, Los Angeles, California 90095-1569, United States.

[†]These authors contributed equally to this manuscript

*For Correspondence. E-mails *lawrensack@ucla.edu*; *maynard@chem.ucla.edu*

Table S1. Results of two-way ANOVAs estimating the effect of drought treatments and presence of either commercial or trehalose gels in the soil on growth and organ allocation. F values and p values are presented for each independent variable for each organ mass. P values associated with significant effects are bolded. We also present the results of the post-hoc Tukey pairwise tests. Significant differences are similarly bolded.

**Trait**	**Treatment F**	**Treatment *P***	**Gel F**	**Gel *P***
model: Trait ~ Treatment + Gel				
Commercial Gel				
Leaf Mass	6.615	**0.00476**	16.261	**4.30 x 10^-04**
Stem Mass	12.71	**0.000141**	35.41	**2.80 x 10^-06**
Reproductive Mass	0.408	0.67211	16.242	**0.00109**
Root Mass	5.347	**0.0113**	22.61	**6.42 x 10^-05**
Trehalose Gel				
Leaf Mass				
Stem Mass	61.72	**1.34 x 10^-10**	12.98	**0.0013**
Reproductive Mass	26.188	**7.36 x 10^-07**	2.653	0.116
Root Mass	6.73	**0.00441**	2.465	0.12852

Post-Hoc Tukey multiple comparisons

	Leaf Mass Commercial	Leaf Mass Trehalose	Reproductive Mass Commercial	Reproductive Mass Trehalose	Root Mass Commercial	Root Mass Trehalose	Stem Mass Commercial	Stem Mass Trehalose
Treatment								
OD-ND	ns	**0.0004**	ns	**0.0000689**	ns	ns	ns	**0.0000013**
TD-ND	ns	**0**	ns	**0.0000004**	ns	**0.038**	**0.005**	**0**
TD-OD	**0.003**	**0.0003**	ns	ns	**0.008**	**0.008**	**2 x 10^-04**	**0.0001119**
Gel								
Gel-NoGel	**0.001**	**0.009**	**1.50 x 10^-02**	ns	**2 x 10^-04**	ns	**0.003**	ns

Table S2. Results of linear mixed models estimating the effect of drought treatments and presence of either commercial or trehalose gels in the soil on leaf water potential, SPAD and stomatal conductance with time. Test statistics and p values are presented for each independent variable and their association with time. P values associated with significant effects are bolded. Stomatal conductance of the commercial treatment was measured once the associated ANOVA testing for an association with treatment and gel presence are presented below as well as the results of Tukey multiple comparisons.

**Model parameter**	**Leaf water potential *t***	**Leaf water potential *p***	**SPAD *t***	***SPAD p***	***g_s t***	***g_s p***
Commercial Gel						
Treatment ND	1.091	0.27944	1.96 x 10^+01	2.00 x 10^-16		
Treatment OD	-2.86	0.00578	0.458	6.47 x 10^-01		
Treatment TD	-2.593	0.01186	0.11	0.9125		
Time	-1.598	0.11553	5.951	1.16 x 10^-08		
Gel	2.044	4.53 x 10^-02	2.198	2.91 x 10^-02		
Treatment OD*Time	2.529	0.01421	-0.935	0.3508		
Treatment OD*Time	1.837	7.14 x 10^-02	-0.427	0.6695		
Gel*Time	-1.583	1.19 x 10^-01	-0.812	0.4178		
Trehalose Gel						
Treatment ND	-0.271	0.787	9.233	2.09 x 10^-15	1.286	0.2009
Treatment OD	-0.946	0.346	1.981	0.0501	-0.392	0.6961
Treatment TD	-0.626	0.532	2.055	0.0422	-0.612	0.5421
Time	-0.891	0.375	2.371	0.0195	2.24	0.0271
Gel	0.39	0.697	-1.87	0.0641	0.235	8.14 x 10^-01
Treatment OD*Time	0.173	0.863	-1.19	0.2364	-0.66	0.5109
Treatment OD*Time	-1.084	0.281	-0.625	0.5333	-1.62	0.108
Gel*Time	0.466	0.642	2.232	0.0277	1.011	0.3144

Trait	Treatment F	Treatment *P*	Gel F	Gel *P*
Stomatal conductance(g_s)	2.216	0.1293	4.378	**0.0463**

Post-Hoc Tukey multiple comparisons

Treatment	
	Stomatal Conductance Commercial
OD-ND	ns
TD-ND	ns
TD-OD	ns
Gel	
Gel-NoGel	ns
